# “It scares me to know that we might not have been there!”: a qualitative study into the experiences of parents of seriously ill children participating in ethical case discussions

**DOI:** 10.1186/s12910-015-0028-6

**Published:** 2015-06-06

**Authors:** Reidun Førde, Trude Linja

**Affiliations:** Centre for Medical Ethics, Institute of Health and Society, University of Oslo, P.O. Box 1130, Oslo, Blindern NO-0318 Norway; Medical Clinic, Oslo University Hospital, Oslo, Norway

**Keywords:** Ethics consultation, Committee, End-of-life, Parents, Discussion

## Abstract

**Background:**

All hospital trusts in Norway have clinical ethics committees (CEC). Some of them invite next of kin/patients to be present during the discussion of their case. This study looks closer at how parents of seriously ill children have experienced being involved in CEC discussions.

**Methods:**

Ten next of kin of six seriously ill children were interviewed. Their cases were discussed in two CECs between April of 2011 and March of 2014. The main ethical dilemma was limitation of life-prolonging treatment. Health care personnel who could elucidate the case were also present in the discussion. The interviewer observed each discussion and then interviewed the next of kin shortly after the meeting, following a structured interview guide.

**Results:**

All next of kin emphasized that it had been important for them to be present. They stressed the important role of the CEC chair and appreciated that their case was discussed in a systematic way. Some next of kin appreciated that the child’s impending death was discussed openly, and believed that this would facilitate their future grieving. Having had an opportunity to hear all the arguments behind the decision to be made would probably help them to accept the road ahead.

All of them felt that they were taken seriously and listened to. They felt that they had added vital information to the discussion. All but one couple did not want any decision-making responsibility, some of them even worried that they might have influenced the discussion too much.

**Conclusions:**

None of the next of kin felt that being present during the CEC discussion had been too heavy a burden. On the contrary, they claimed that their presence in a CEC discussion may add vital information to the discussion and may improve the quality of the decision. It is important that the CEC’s role is explained to them so they are well prepared for what to expect. They need to be followed up after the discussion.

## Background

Clinical ethics committees (CEC) can function as an aid for clinicians in ethically difficult decisions [1]. In Norway, all health care trusts must have a CEC, and the first committees were established in 1996. Norwegian CECs have an average of ten members [2]. All have medical doctors, nurses and a hospital chaplain as members. In addition, the majority include a member with legal knowledge, a member with ethics competence, and a patient representative [2].

The CEC’s aim is to ensure open and thorough elucidation of a problem in which the opinions, values, and interests of all involved parties are included [3]. According to Norwegian law, the patient’s treating physician has the final responsibility for the medical decision [4]. Usually, key staff members involved in the treatment and care of the patient are present in the CEC discussions. In addition, a few committees now routinely invite patients / next of kin into the committee’s discussion [5, 6], though they do not always accept the invitation. These committees find that the participation of patients and next of kin in the discussions adds important, sometimes vital information, and that the description of the situation given by the clinicians may not be sufficient for a thorough and balanced discussion [6, 7].

In order to cover relevant aspects of an ethically complex dilemma, most of the CECs follow a structured discussion model (Table 1) which is modified according to the case.

**Table 1:** The discussion model for a structured CEC discussion

● Which ethical problem(s) is (are) involved?
● Clarification of the facts of the case (medical, psychosocial).
● Identification of all involved parties and their (possible) conflicts of interests.
● Identification and clarification of values, principles, and virtues at stake, relevant guidelines and legal issues.
● Discussion of possible solutions and their consequences.
● Evaluation/follow up.

In order to critically scrutinize the quality of the CECs’ work, regular and systematic evaluation is necessary [8, 9]. Do the CECs work in compliance with the guidelines which society has set up, and in particular, are the patients’ perspectives and interests properly safeguarded in the case discussions [3]?

Patient values and opinions can be included in a case discussion in several ways: by having a patient representative as a committee member, by including a specific member of the team who represents the patient in the discussion, or by conducting a CEC interview with the patient/next of kin prior to the discussion [1, 10]. Finally, the patient/next of kin may be invited to participate in the discussion [1, 5, 6]. It is our impression that one common reason for not including patients/next of kin in the CEC discussion may be that it is seen as a heavy emotional burden to be confronted with a group of strangers who discuss deeply personal matters. Another reason may be that clinicians and CEC members may be reluctant to share information and opinions openly in their presence, in particular if there are conflicts involved in the case. If so, the quality of the discussion may suffer.

Parents of seriously ill children may be particularly vulnerable. In addition to being their child’s legal guardian, they may struggle with their own feelings of grief and anxiety. It may be seen as impossible to include them in a case discussion in a way that protects their feelings and interests. Getting feedback from patients/next of kin who actually have participated in such discussions is important in order to weigh the balance between benefits and costs of their involvement, and to learn how the process can be improved so that their specific feelings and needs are taken care of in the best possible way [11]. We have found little empirical knowledge about this in the literature [12]. One exception is a study of 20 ethics consultations done in 1990–1992 by Mc Clung *et al.* which found that patients/relatives were less satisfied with the consultations than physicians and nurses [13]. However, these consultations were mainly done with consultants and small teams, not in a committee. Accordingly, we feel it vital to gain knowledge of parents’ experiences of being actively included in life and death discussions in a CEC.

## Methods

The study which took place between April of 2011 and March of 2014 is based on interviews with ten next of kin following their participation in seven case discussions in two CECs. The two CECs have physicians as chairs, a legal expert, an ethicists and a patient representative as members. The two committees are the most experienced among the Norwegian committees who include patients/next of kin in the CEC discussions.

All external participants who meet with these committees receive a written letter before the meeting explaining the role of the CEC, and stressing that the committee does not have a decision-making authority. After a presentation of the CEC’s mandate and a short presentation of the people in attendance, the chair invites the clinicians to describe the ethical dilemma(s) and the medical and psychosocial situation (*e.g.* different specialists, nurses, physiotherapists, psychologists). The next of kin are then invited to give their portrayal of their child and of their family’s situation.

The included CEC discussions concerned dilemmas regarding limitation of life-prolonging treatment of seriously ill children, where the children’s next of kin had participated in the discussion, and which were brought to the knowledge of the first author during the study period. No eligible cases were excluded, and all next of kin who were asked to be interviewed, accepted.

The sample consists of six interviews, one grandfather who was actively involved in his grandchild’s life and who represented the parents, and nine parents of five children. One case was discussed twice, three months apart. All cases were referred to the CECs by the children’s doctors.

T.L. (a medical student, and a non CEC member) conducted the interviews. For practical reasons this took place right after the CEC meeting, with one exception where the interview took place three days after the CEC discussion. In order to understand more of the dilemmas, and to be able to interpret the answers in a context, the interviewer also observed the discussions and took notes. The observation focused on the communication between the next of kin and the committee members, the next of kin’s reactions during the meeting, and how the CEC members responded to these reactions.

The doctors responsible for the treatment of the child contacted the parents before the CEC discussion and asked whether they were willing to 1) have the interviewer (TL) present as an observer during the CEC discussion, and 2) to be interviewed by her at a time which would be convenient for them. It was emphasized that the aim of the study was to improve the way patients and next of kin are included in CEC work. The next of kin gave their informed consent (written and oral) to be interviewed. In the case discussed twice, the interview took place after the second discussion. In both these discussions the patient, an infant, was present.

The seven discussions were chaired by four different CEC members.

Except for the parents of one child, all interviewees were native Norwegians. The non-Norwegian parents were assessed to have acceptable Norwegian language skills, but to secure understanding, an interpreter was present in both case discussions, as well as in the interview.

The interviews followed a structured interview guide (see Table 2). The interviewees could talk freely in length or give short, concise answers. Thus, the interviews lasted between 20 and 45 min. The interviews were tape recorded and transcribed verbatim by TL.

**Table 2:** Interview guide

1. Looking back, how did you experience the CEC meeting?
2. Were you positive or ambivalent about participating in the CEC discussion?
3. Can you describe the preparatory information which you received before the CEC meeting?
4. Was the information you received in advance in accordance with what actually happened?
5. Did the committee give concrete advice about what was the right thing to do?
6. Did you receive any information in advance about who would be present in the meeting?
7. Thinking back on the persons present in the meeting
a) Were too many people included?
b) Anyone you feel should have been present, but who was not?
8. How do you feel that your “case” was discussed?
a) Do you feel that the case became better illuminated through the discussion?
b) Did the discussion help you accept the final decision?
9. Did you understand what was being said in the discussion?
10. Did new, surprising, or disturbing information come up during the meeting, and if so can you describe how you reacted?
11. Did you feel that you were listened to, or was it difficult to speak?
12. Now thinking back, are you pleased to have participated in the discussion?
Finally, do you have anything to add?

The two authors have read all the interviews several times. The text has been analyzed thematically according to the interview guide, but with a special focus on salient expressions on themes not included in the interview guide. The analysis is inspired by Kvale and Brinkmann’s term “bricolage” meaning that we have moved back and forth in the written material without relying on one particular method or analytical technique [14]. In the analysis we have had a special focus on critical comments and suggestions for improvements.

After the results section was written, we have gone back to the original text to double check our interpretations and to ensure that the citations had not been detached from their context.

### Ethical considerations

Due to anonymity considerations the cases are not described in detail, nor are personal characteristics of the interviewees.

According to the Health Research Act (ACT 2008-06-20 no. 44: Act on medical and health research) this project did not need to be submitted to a research ethics committee. The project has been approved by Norwegian Social Science Data Services (NSD).

## Results

All case discussions involved children who had been seriously ill for months or years, and who, without the intervention discussed, most likely would die within days/a few months.

Although none of the cases involved open conflicts concerning the decision, in one of the cases the parents felt that the child had been suffering needlessly during long term intensive care treatment, while the doctors in charge felt that stopping intensive care treatment might be too early, and accordingly requested a CEC discussion. In another case, the parents feared that life-prolonging treatment could be withheld too early. In a third case, the parents requested experimental treatment while the doctors were uncertain whether that would be in the child’s best interest.

During the CEC discussions all next of kin were active and deeply engaged in the discussions. Although some cried and showed grief and frustration, they all seemed to cope well during the meeting. They contributed to the discussions with reflections regarding the child, the child’s siblings, and their family’s situation in an open and straightforward way.

The CEC members responded with verbal and non-verbal sympathy. Before closing the meeting a brief summary of the discussion was made by the committee chair.

### Being part of the discussion was important, but overwhelming

All interviewees claimed that they had never been in doubt that they had wanted to be present during the CEC discussion. All interviewees felt that it had been right for them to participate. One couple was surprised, but very pleased to be invited into the discussion.

*We were surprised that we could participate,- that our opinion counts. And that they wanted to learn what we think and that they wanted to meet our child. And that she was important- very good! (I-6)*

*This discussion should have taken place earlier- it would have helped me in my grieving (I-2).*

*It means a lot to be allowed to participate, to be invited in, a report would never do the same (I-3)*

They claimed that being invited to be present in the discussion increased their confidence in the health care system. The CEC is part of this system, and they appreciated that their voice mattered.

All interviewees stressed that they had felt that their presence was extremely important in order to obtain a correct picture of the situation.

*We are here at least 23 h a day, we see much more (of the child), both because we are around a lot more, but also because we have a different relationship with the child, compared to when the white coat enters wanting a particular response, which he often does not get in the three minutes he is there, and then he claims that there is no response, which is wrong because we who are there, see a response. (I-1)*

The parents claimed that the doctors’ descriptions of the child had been biased and incomplete. This included the medical information presented by the doctors, a too pessimistic prognosis and above all the biased descriptions of the children and the children’s awareness.

*I really do not understand how you could have grasped the whole picture based only on what the doctors said! (I-4)*

*If I had not been asked (to participate) and I had known that there actually was a meeting, and none of us had been there, I would not have been very pleased. (I-5)*

*..Here they (the neurologist and neurosurgeon) were presenting facts based on a number, it is not a person, and with a negative focus: “this is very serious brain damage and there has been no progress.” I had a feeling that they were describing a case, I had an urge to show a picture of my child, and I could have supplemented with a video and showed communication which gives a completely different impression. I think many got a picture of a vegetable…(..) it was disappointing and insufficient. In fact it scares me to know that we might not have been there. (I-1)*

One couple said that the doctors neglected and underreported their child’s obvious suffering, and that it was vital that the CEC members became aware of this through the parents’ description.

Although they met a large group of strangers who discussed matters of vital importance to them and their families, and although it was an overwhelming experience, all interviewees looked back on the meeting with few critical comments:

*Unreal, like participating in a movie, overwhelming, important to participate, did not exactly know what was going to happen. (I-1)*

*A bit stressful, but also useful. (I-6)*

*It was ok,.. didn’t exactly know what to expect, but I experienced a calm atmosphere, people who were listening to what I could tell them. (I-2)*

*And, I felt it was a good way to include parents in this process. I experienced it as a dignified meeting, yes a good meeting. I see it as a good process. (I-3)*

*Balanced, honest, open, nobody had their private agenda… People were honest and respected each other (I-5).*

None of the interviewees felt that too many people were included in CEC discussion. On the contrary, they mentioned people who were not there, whom they would have liked to see, *e.g.* the hospital chaplain, a nurse or physiotherapist who knew the child well, or an (absent) ethicist. Several of the interviewees appreciated the presence of an external medical ethicist, and some said that it was positive to have people present who did not wear the hospital uniform.

In addition to making sure that the CEC members could “see” their child as a human being, the next of kin appreciated being seen, having their situation acknowledged, and receiving sympathy and support from the committee members.

*All participants managed to keep their heads cool enough, while at the same time they were warm people, it is interesting. (I-5)*

Several of the interviewees said that they appreciated the systematic structure which was used during the case discussion. The important role of the CEC chair was underlined. That the chair showed sympathy was appreciated. In addition to being responsible for the structure of the discussion, it was appreciated that the chair made sure that all participants were heard.

### Sharing information is important

Although none of the interviewees felt that the discussion brought up new information, they emphasized that hearing the case presented from several angles and without the usual time restrictions, resulted in medical information being perceived in a different way.

*One thing is being invited to a consultation with the doctor, quite another matter is having the feeling of being listened to. (I-3)*

Several interviewees said that it was useful to have the information commented on by many people with different professional backgrounds. All interviewees emphasized the importance of the varied professional backgrounds of the CEC members, *e.g.* an oncologist who could analyze their case from a more distanced, but at the same time knowledgeable perspective.

*Things became clearer in the sense that we have obtained an improved understanding of the options…(..). ..We (already) have agreed that this is where we have to go from here, these are the plans. But now the case was discussed by people with a lot of experience, who have the ability to understand both the human and the medical aspects of our case. Which I think is very good. (I-3)*

Through the open dialog the ethical problem was illuminated from several angles.

*And then you have the possibility to pose follow-up questions: What exactly do you mean? What do you think about this, and why? (I-1)*

One topic brought up in the discussion that was mentioned by the interviewees, and which according to them, had not hitherto been mentioned in the hospital setting, was the child’s impending death. This was much appreciated. One couple felt relieved that the committee would focus on how the child would die, and what could be done to relieve the child’s suffering during the dying process.

*Do you know, I think like this: To have the chance to talk about the fact that a child will die. It has been hidden. (I-3)*

*Now I know that ok, now we have this time left. Then you mobilize. (I-3)*

### Participation made it easier to accept the decision

Several of the interviewees said that although the discussion did not end up with a distinct conclusion about what to do, they felt that a conclusion actually had been reached, but in a non-explicit way.

In the cases where a conclusion was reached, and the decision was based on a consensus in the group discussion, the next of kin were confident that this was the right (although painful) decision. Further, when a conclusion was being formulated, *e.g.* that it was not in the child’s best interest to continue aggressive life-prolonging treatment, some next of kin felt relieved, and some said that the conclusion was expected (although dreaded), but that it was easier to accept after they had heard all pros and cons from the specialists themselves.

*We could hear and listen to what the pediatric surgeon said. Much preferred to having it referred to by others. (I-3)*

*You understand the options….Then you conclude. Then we parents at least understand the conclusion, and why this was the chosen path. (I-3)*

*…Although I disagreed, I understand why they (the clinicians) have this opinion…. (I-1)*

Although one couple had insisted that they should have the final say, and in advance had regarded the CEC discussion as unnecessary, being present in the discussion had made a difference.

*After this meeting we perhaps realize that things will not develop as positively as we have hoped, … It is thanks to these two meetings, but also to the fact that time has passed, that we now think a little differently, and we have realized that we must listen to the doctors and to their arguments. (I-6)*

Two of the couples emphasized the importance of including the CEC’s conclusions in the medical record so that new clinicians who had not participated in the CEC discussion could be made aware of them. These parents were not confident that the value assessments made by the CEC would be followed up, and said that they wanted a new CEC discussion if new dilemmas came up. They did not want the doctors to make a serious decision based on their own evaluation alone*.*

### Parents should be allowed to be just parents

With the exception of one couple, none of the next of kin wanted a greater say in the serious decisions that had to be taken. They did not want the decision-making responsibility. Two parents even worried that they perhaps had dominated the CEC meeting too much, that they had been allowed to talk too much.

*It is a scary thought that we have so much power if we want something and go for it…And if you later find that the decision was wrong, you will never forgive yourself. (I-1)*

*It is very important to us that the final decision is not ours…This is very important, so we can just be parents. (I-4)*

### Routines must be improved

Although they received written and oral information, a general impression was that the next of kin were not well enough prepared for the meeting, and in particular did not know the role of the CEC. Thus, one couple had misunderstood the purpose of the meeting, thinking that the committee would decide the child’s future treatment.

Several interviewees would have liked to know in advance who would be present in the discussion. One couple had found the names of the CEC members and googled them. Another couple had discussed whether they should prepare for the meeting, but decided to relax and see what would happen. Being well enough prepared was emphasized as being particularly important for people not used to talking to a large group of people.

*One may easily feel small I believe, parents may feel like “who am I here.” Because it was overwhelming to enter the room, although we discovered that they were very humble, it may be a scary experience for some. (I-1)*

Some also said that the chance to talk after the meeting, in the research interview, had been useful for them because they then could elaborate on what they had experienced in the meeting.

The importance of translating medical terminology into understandable terms was emphasized by several of the interviewees. The parents, who were not native Norwegians, emphasized that the discussion in itself was complicated. They suggested that when next of kin are not native it is better to assume that they do not speak the language at all and to have everything translated. They also felt that language difficulties prevented them from expressing everything they had wanted to say.

The interviewees criticized the fact that the existence of an ethics committee had not been known to them in advance, and stated that the existence of a hospital CEC should be better known by patients/next of kin.

Although the interviewees appreciated that their case was discussed in a systematic and understandable way, one couple criticized that the ethical analysis of the case was underdeveloped. This couple felt that the discussion was too much dominated by numbers and discussions of prognosis.

*NN talked a lot about the medical facts, if one does this, that will not happen…(..), and this should not be the only focus of interest, the CEC- members should help lift the debate up on a higher level (…) and bring in new perspectives, one is human dignity, suffering, what is it? (…) There are a lot of such things that are fundamental and which could have been developed more, what is good and bad, and what is right and wrong, based on the individual. (I-1)*

## Discussion

The present study illustrates that it may be important to invite next of kin into ethics discussions of serious end-of-life decisions for several reasons. Ethics consultation services’ raison d’etre is to provide a discussion of pros and cons based on a balance of the perspectives [7]. In most of these ethical dilemmas, the information given by the people who are most involved is vital [11]. Being present to hear all information presented to the CEC by health care personnel and being allowed to add their own version of the child’s situation to the discussion was perceived both meaningful and important by the next of kin. Although only ten next of kin were included, the findings also reveal room for improvement in routines when patients/next of kin are invited into ethics consultations.

Great care should, however, be taken not to regard this study as presenting a final truth. We have used an interview guide with few open questions which is at variance with the recommendations for a qualitative study [14]. We wanted to hear the study participants’ view on points of improvement in the committees’ routines. In addition, we wanted the next of kin’s reaction to arguments for not including patients/next of kin in CEC discussions, *e.g.* that it is too overwhelming to meet so many strangers and that new and disturbing information will upset them. The questions we posed gave meaningful information within limited use of time. Therefore the questions may be used in a future national survey. Further, the study has taken place in a Norwegian setting. Most of the studies being done on patients and next of kin’s evaluation of ethics consultations are done in the US, and the settings have been individual consultations and small teams [11]. We have studied next of kin of children. As legal guardians parents have a special role in protecting the patient’s interest. Thus, the results do not necessarily apply to next of kin of adult patients. Add to this that the persons in our material were deeply involved, updated on medical information, and with strong opinions and intellectual resources. They are not necessarily representative of all next of kin in similar situations.

It may not feel right for all next of kin to participate in a CEC discussion. In such cases they should be allowed to appoint a person among the health care personnel/from the staff who knows them well. Further, not all CEC discussions are prior to serious decisions. Some discussions are retrospective, and in some instances doctors may wish to discuss a possible upcoming situation. In the latter cases it may be too early to involve patients/next of kin. Further, it is important that the committees, who invite patients/next of kin into the discussion, have the competence to treat both the next of kin and the participating clinicians in a competent, balanced and fair way.

Both CECs studied here are experienced. But experience does not guarantee that the discussion is without problems. A routine follow-up conversation with the next of kin may be useful for them, and may above all give information to improve the work of the CEC.

The belief that it is too heavy a burden for next of kin to participate in ethical discussions of life and death issues, was not supported in this study. On the contrary, the interviewees were concerned that more patients and relatives should know about this possibility. Our next of kin had been living with the impending death of their child for months and years, and some were even relieved that death was finally brought up as a topic in the hospital setting. There are lessons to be learned from this far outside a CEC setting.

The presence of patients/next of kin may be useful for several reasons. First and foremost it secures that relevant aspects of the case are illuminated. The interviewees felt that their voice in the discussion had been absolutely necessary, particularly to provide a balanced portrayal of the child’s personality, functioning, and quality of life. Seeing and hearing the individuals and their narratives is not the same as having it referred (not always correctly) by health care personnel [6]. It is likely that this information improves the quality of the discussion and the normative conclusions. Observation of communication between the next of kin and doctors and nurses is useful to increase the CEC members’ understanding of the ethical difficulties and the conflicts [6].

When information is presented in a group discussion, it may be processed differently; misunderstandings and misconceptions can be uncovered, as pointed out by our interviewees. This is the most important reason for including next of kin in the discussion. Honest and complete information is important to parents of seriously ill children [15–17]. This may also increase trust and the understanding of the reasons for (often dreaded) decisions. Trust may be increased if next of kin feel that they are being taken seriously, as all our interviewees stated. McClung *et al.* found that when families were dissatisfied with the ethics consultation, they were more likely to be dissatisfied with the health care personnel’s communication [13]. Our next of kin described how well-intending clinicians already had presented vital information which, however, had not been fully understood. The words used might have been correct, but they had a different effect when the same information was given with no time stress, when asking and listening is encouraged, and doubts can freely be expressed. A well-functioning CEC may thus be a forum where misunderstandings may be detected, and unproductive communication may be improved so that the decision-making process can get back on track within the team [6].

It is also thought-provoking that many of the interviewees were afraid that they had been given too much of a voice in the discussion. Most of the parents feared that having decision-making power in these decisions could make them responsible for the death of their children.

### Points of improvements

Having patients and their representatives as part of a CEC discussion is a new endeavor, and we must take care to learn to do this in a competent and considerate way. The knowledge of the existence of an ethics consultation service in the hospital is limited among the public. Patients and next of kin must be informed about the CEC, its role and mandate, and how to get in touch.

In addition to a written presentation of the purpose of the discussion, the members could be presented by photo and affiliation. It might be useful for the committee chair to meet with the patients/next of kin before the discussion to supplement the written information and to reduce the level of anxiety.

The background of the committee members, how the CEC will discuss their case, and what is expected of them as next of kin should be part of the preparatory information before each discussion. Using a non-medical language is also important.

## Conclusions

The present study illustrates that it may be important to invite parents of seriously ill children into clinical ethics committees’ discussions of difficult end-of-life decision. Being present to hear all information presented to the CEC by health care personnel and being allowed to add their own version of the child’s situation to the discussion were perceived as both meaningful and important by the next of kin. They felt taken care of, and listened to. It is important that the next of kin receive good information in advance of the CEC meeting and preferably also a follow-up call.
